# Temporal changes in cardiac oxidative stress, inflammation and remodeling induced by exercise in hypertension: Role for local angiotensin II reduction

**DOI:** 10.1371/journal.pone.0189535

**Published:** 2017-12-12

**Authors:** Sebastião D. Silva, Zaira P. Jara, Roseli Peres, Larissa S. Lima, Cristóforo Scavone, Augusto C. Montezano, Rhian M. Touyz, Dulce E. Casarini, Lisete C. Michelini

**Affiliations:** 1 Department of Physiology & Biophysics, Biomedical Sciences Institute, University of Sao Paulo, Sao Paulo, SP, Brazil; 2 Institute of Cardiovascular and Medical Sciences, BHF GCRC, University of Glasgow, Glasgow, United Kingdom; 3 Department of Medicine, Federal University of Sao Paulo, Sao Paulo, SP, Brazil; 4 Department of Pharmacology, Biomedical Sciences Institute, University of Sao Paulo, Sao Paulo, SP, Brazil; Albany Medical College, UNITED STATES

## Abstract

Exercise training reduces renin-angiotensin system (RAS) activation, decreases plasma and tissue oxidative stress and inflammation in hypertension. However, the temporal nature of these phenomena in response to exercise is unknown. We sought to determine in spontaneously hypertensive rats (SHR) and age-matched WKY controls the weekly effects of training on blood pressure (BP), plasma and left ventricle (LV) Ang II and Ang-(1–7) content (HPLC), LV oxidative stress (DHE staining), gene and protein expression (qPCR and WB) of pro-inflammatory cytokines, antioxidant enzymes and their consequence on hypertension-induced cardiac remodeling. SHR and WKY were submitted to aerobic training (T) or maintained sedentary (S) for 8 weeks; measurements were made at weeks 0, 1, 2, 4 and 8. Hypertension-induced cardiac hypertrophy was accompanied by acute plasma Ang II increase with amplified responses during the late phase of LV hypertrophy. Similar pattern was observed for oxidative stress markers, TNF alpha and interleukin-1β, associated with cardiomyocytes’ diameter enlargement and collagen deposition. SHR-T exhibited prompt and marked decrease in LV Ang II content (T_1_ *vs* T_4_ in WKY-T), normalized oxidative stress (T_2_), augmented antioxidant defense (T_4_) and reduced both collagen deposition and inflammatory profile (T_8_), without changing cardiomyocytes’ diameter and LV hypertrophy. These changes were accompanied by decreased plasma Ang II content (T_2_-T_4_) and reduced BP (T_8_). SHR-T and WKY-T showed parallel increases in LV and plasma Ang-(1–7) content. Our data indicate that early training-induced downregulation of LV ACE-AngII-AT1 receptor axis is a crucial mechanism to reduce oxidative/pro-inflammatory profile and improve antioxidant defense in SHR-T, showing in addition this effect precedes plasma RAS deactivation.

## Introduction

Hypertension is a major reversible risk factor for cardiovascular disease worldwide [1]. Hyperactivity of the renin-angiotensin system (RAS) with imbalance between ACE-Ang II-AT1 receptor axis and its counter regulatory ACE2-Ang-(1–7)-Mas receptor axis is a hallmark of chronic hypertension leading to functional deficits and structural organ damage [2–7]. Several studies have shown that high circulating and tissue Ang II levels are potent stimuli to increase reactive oxygen species (ROS) and pro-inflammatory cytokines’ expression that further activate oxidative stress and the expression of several RAS components, perpetuating a deleterious positive feedback mechanism [4,5,8–10]. In the heart, these hypertension-induced responses are accompanied by fibrosis, myocardial hypertrophy and deleterious cardiac remodeling [11,12].

Accumulating experimental evidence has also shown that exercise training, a non-pharmacological tool, is very effective in improving autonomic control, downregulating RAS, reducing oxidative stress and inflammation, thereby contributing to blood pressure lowering [4,5,7,8,10,13–15]. Although regular physical activity is highly recommended for the treatment of hypertension [1,16,17], the precise mechanisms by which exercise training improves cardiovascular control are not completely understood. Increasing evidence has shown that hypertensive hearts respond to exercise training with reduced ROS availability, increased antioxidant capacity, decreased inflammation and reduced fibrosis [8,17–21]. However, some controversial effects have been described in RAS expression/activity in the LV and plasma of trained rats. Gomes Filho et al (2008) described decreased plasma Ang II without Ang-(1–7) content changes and increased LV Ang-(1–7) levels and MAS receptor expression without changes in tissue Ang II levels. Fernandes et al (2011) confirmed the increased Ang-(1–7) levels and augmented ACE2 activity in the trained heart but reported reduced LV ACE expression/activity with diminished Ang II levels in the presence of increased AT1 receptor expression. In general, these observations were made at the end of an exercise training protocol. To our knowledge there is no information on the sequential changes driven by exercise on heart structure and function and little is known about differential responses between normotensive and hypertensive conditions. Since previous studies showed that plastic changes in brain autonomic areas preceded exercise-induced functional responses [10] and that training-induced downregulation of the RAS in the brain and vessels is a prompt response with changes in precursor and receptors occurring at different time span [4,7], we hypothesized that exercise would differentially affect hormonal control and LV structure and function. Therefore, in the present study, we analyzed in spontaneously hypertensive rats (SHR) and age-matched normotensive controls the temporal effects of aerobic training on plasma and LV Ang II and Ang-(1–7) content. Additionally, we analyzed in the LV of both strains the time course changes induced by training on oxidative stress, pro-inflammatory profile, antioxidant defense and their consequence on hypertension-induced cardiac remodeling.

## Materials and methods

All surgical procedures and experimental protocols used were in accordance with the Ethical Principles in Animal Research adopted by the Brazilian College of Animal Experimentation, and were approved by the Institutional Animal Care and Use Committee of the University of São Paulo (CEUA, protocol number 188; page 115, Book 2).

### Animals and training protocols

Male SHR and WKY rats, aged 11–12 weeks at the beginning of the protocols, were obtained from colonies maintained at the Institute of Biomedical Sciences of the University of São Paulo, Brazil and housed in the Animal Facilities of the Department of Physiology & Biophysics, Biomedical Sciences Institute at a controlled room temperature, with a 12:12 h light:dark cycle and free access to tap water and food. Rats preselected for their ability to walk/run in a treadmill (Inbramed, KT-300, Porto Alegre, Brazil) were submitted to graded exercise testing on a flat treadmill starting with 0.3 km/h, with increments of 0.3 km/h every 3 min, up to the exhaustion, defined as the moment the rat stopped running, lying on the treadmill floor. Maximal exercise tests were used to calculate the intensity of aerobic training (T, 50–60% of maximal exercise capacity). Rats with equal performance were allocated to low-to-moderate T protocol (running sessions of 1 h/day, 5 days/week, 0% inclination) or kept sedentary (S) during 8 weeks [7]. Maximal exercise tests were repeated at weeks 4 and 8 to adjust training intensity and quantify the training effects at the end of protocols, respectively. We excluded from the analysis rats that did not run or stopped running during the T protocol.

### Arterial catheterization and cardiovascular recordings

At established time points (weeks 0, 1, 2, 4 and 8 for T-groups and weeks 0 and 8 for S groups), rats (12-18/sub-group) were anesthetized with ketamine plus xylazine (100 mg.kg^-1^ + 20 mg.kg^-1^, *ip*.) for chronic catheterization of the femoral artery. Rats were treated with antibiotic and analgesic and allowed to recover for 1 day. Resting values of pulsatile arterial pressure (AP) and heart rate (HR) were recorded in conscious freely moving rats on the following day, at least 24 hours after the last training session [10]. AP was acquired on a beat-to-beat basis for ~40 min (2 kHz sampling frequency, IBM/PC computer, Lab Chart Pro, AD Instruments, Bella Vista, Australia) after the stabilization of cardiovascular parameters. HR was determined from pulse interval between two systolic peaks.

### Blood and tissue sampling

After functional measurements rats were deeply anesthetized (sodium pentobarbital 60 mg.kg^-1^ *ip*.) and the thorax was opened to access the heart immediately after the respiratory arrest. Two ml of blood was taken from the LV with a heparinized syringe. Rats were then submitted to transcardiac perfusion with KCl 14 mM (heart was stopped in diastole) diluted in sterile saline for 5 minutes. The heart was removed; the LV was isolated, weighed and cut transversely in the middle. Half of the LV was fixed for 24 h in formalin 4% and stored in alcohol 90% for morphometric analysis. The other half was stored at -80^o^ for biomolecular studies or immersed in tissue freezing medium for analysis of the oxidative stress.

### Angiotensin measurement

Angiotensins were extracted from the LV and plasma as previously described [22,23]. LV tissue was weighed and homogenized with 100 mmol/L PB, pH 7.2, containing 340 mmol/L sucrose, 300 mmol/L NaCl and a mix of proteases inhibitors (Complete Mini Roche). Protease inhibitors were also added to plasma (50 μl in 500 μl). Samples were centrifuged at 15,000 rpm, 4°C during 20 minutes and then concentrated in C18 Sep-pak columns previously activated with methanol (5 ml), tetrahydrofuran (5 ml), hexane (5 ml), methano (5 ml) and water (10 ml). Using ethanol, acetic acid and water (90:4:6) the peptides were then eluted. Next, the elutions were lyophilized and suspended in 500 μl of mobile phase A: 5% acetonitrile (50 ml) in 0.1% orthphosforic acid (1 ml). The peptides were separated in a reverse-phase B: 95% acetonitrile in H_3_PO_4_ 0.1% in a flow of 1.5 ml/min during 40 min in the Milton Roy System, constituted of two constaMetric 3000 pumps, a UV detector spectral Monitor 3100, a programmer GM 4000 and a mixer. Reverse phase HPLC was used to measure the angiotensins. Synthetic standards were used and peptide detection was carried out at 214 nm. The results were corrected by weight or volume and angiotensins’ content were expressed in pmol/g (LV) or pg/ml (plasma).

### Quantitative real-time PCR

mRNA expression was analyzed by the qPCR, as described previously [10,24]. Briefly, total RNA was extracted using TRizol^®^ reagent according to the manufacturer’s instructions (Invitrogen Life technologies, CA, USA) and measured by NanoDrop Spectrophotometer (Nano-Drop Techonologies, USA). Total RNA was treated with RNase-free DNAse I (Invitrogen Life Technology) and 2 μg of RNA were reverse transcribed in a reaction containing oligo-dT (100 μg/mL), 10 mmol/L of 2′-deoxynucleoside 5′-triphosphate, 5× First-Strand buffer, and 2 μL of 200-U Moloney Murine Leukemia Virus (M-MLV) reverse transcriptase (Invitrogen Life Technology). Two μL of each reverse transcription product was amplified in a reaction buffer containing 5 μL of SYBR Green PCR master mix (Applied Biosystems) and 900 nmol/L of one of the primers listed in the Table 1, at a final volume of 10 μL per sample. The reaction conditions consisted of 40 cycles (2 min at 50°C and 2 min at 95°C, 15 s denaturation at 95°C, 60 s annealing at 60°C). The relative mRNAs’ expression (target gene/reporter gene) was analyzed by ΔΔCt method [25] and reported as arbitrary units. Hypoxanthine phosphoribosyl transferase (HPRT) was used as the reporter gene.

**Table 1:** Sense and antisense sequences of primers used.

Gene	Forward Primer	Reverse Primer
Sod1	TTGGAGACCTGGGCAATGT	TCCACCTTTGCCCAAGTCA
Nqo1	TCAGCGCTTGACACTACGA	TCTTCAGAGCCTCCACAGC
Prdx1	CTTCCCACCCTCCCTGAAG	CCCAGTTCCCGCAGACTTA
Txn	AGACGTGGATGACTGCCAG	GCACCAGAGAACTCCCCAA
Ho-1	TGGCACATTTCCCTCACCA	GCCTCTACCGACCACAGTT
Catalase	GGCTCACACACCTTCAAGC	TGTGCAAGTCTTCCTGCCT
Gpx	AGTGCGAGGTGAATGGTGA	ACTTGGGGTCGGTCATGAG
TNF alpha	TGCCTCAGCCTCTTCTCATT	CCCATTTGGGAACTTCTCCT
Il1b	CTGTGACTCGTGGGATGATG	GGGATTTTGTCGTTGCTTGT
Il10	CCTGCTCTTACTGGCTGGAG	TGTCCAGCTGGTCCTTCTTT
Myh6	CACCAACCTGTCCAAGTTCC	ATCGTGGATTTTCTGCTTGG
Myh7	AAACTGAAAACGGCAAGACG	TGACGGTGACACAGAAGAGG
Nppa	AGGGCTTCTTCCTCTTCCTG	CCAGGTGGTCTAGCAGGTTC
Acta	GTCGGTATGGGTCAGAAGGA	TGTCGTCCCAGTTGGTGATA
Col1a1	TTGACCCTAACCAAGGATGC	CACCCCTTCTGCGTTGTATT
Col3a1	AACGTGGCTCTAATGGCATC	CATCTTTTCCAGGAGGTCCA
Hprt	TTTTGCTGACCTGCTGGATTAC	TACTTTTATGTCCCCCGTTGA

Genes are named according to the official nomenclature on NCBI gene database. Sod1, superoxide dismutase 1; Nqo1, NAD(P)H dehydrogenase quinine; Prdx1, peroxiredoxin; Txn, thioredoxin; Ho-1, hemioxigenase 1; Gpx, glutathione peroxidase; TNF alpha, tumor necrosis factor alpha; Il1b, interleukin-1β; Il10, interleukin-10; Myh6, myosin heavy chain, α isoform; Myh7, myosin heavy chain, β isoform; Nppa, atrial natriuretic peptide; Acta, α-actin; Colla1, collagen I; Col3a1, collagen III; Hprt, hypoxanthine phosphoribosyl transferase.

### Catalase activity determination

Catalase activity was measured by a colorimetric assay using Amplex Red Catalase Assay Kit (Molecular Probes) according the instructions provided by the manufacturer. Briefly, hearts were homogenized in lysis buffer (50 mM Tris, pH 8.0, 150 mM NaCl, 1% Triton X-100, 0.1% SDS), supplemented with 1 mM phenylmethylsulfonyl fluoride, 1 μg/ml pepstatin A, 1 μg/ml leupeptin, 1 μg/ml aprotinin (Sigma-Aldrich), 10 mM sodium fluorate (AnalaR Normapur; VWR, Leuven, Belgium), and 1 mM sodium orthovanadate (Alfa Aesar, Ward Hill, MA). Supernatants were transferred to microtubes and protein concentrations were determined using the DC protein assay kit (Bio-Rad Laboratories, Hercules, CA). For the assay, 15 μg of total protein were used.

### Detection of reactive oxygen species (ROS) in the left ventricle

The oxidation-sensitive fluorescent dye dihydroethidium (DHE) was used to evaluate ROS content *in situ*, as previously described [26]. LV embedded in tissue freezing medium and frozen was cut in a cryostat (Leica CM 1850; Nussloch, Germany). Transverse LV sections (14 μm) were disposed on glass slides and allowed to reach the equilibrium (30 min at 37°C in phosphate-buffered saline, PBS). The sections were then incubated with DHE (50 μM DHE in PBS; Sigma Aldrich) at 37°C for 30 min in the dark; control sections received an identical volume of PBS. Fluorescent images were subsequently obtained with an optical microscope (200x magnification, Leica DFC 300 FX, Wetzlar, Germany). Both total and background pixel intensity were measured and used to correct the DHE intensity of each image. Quantification of DHE intensity was carried out using ImageJ software (Wayne Rasband, National Institutes of Health, USA) and expressed in arbitrary units.

### Morphological and morphometric analysis in the LV

Cardiac hypertrophy was evaluated in SHR and WKY groups during T and S protocols by the LV weight / tibia length ratio and the relative fibrosis area. It was confirmed at the end of protocols by the measurement of myocytes’ diameter. For this proposal, tissue was immersed in 10% buffered formalin and fixed for 24 h. LV was embedded in paraffin, cut into 5 μm sections at the level of the papillary muscle and subsequently stained with either hematoxylin-eosin (HE for cellular structure visualization and measurement of myocytes’ diameter) or picrosirius red (to measure the relative fibrosis area). Under visual inspection (KS 300, Zeiss light microscope with 400X or 200X magnification for myocytes or fibrosis quantification, respectively) 2 randomly selected sections from each animal were acquired and quantified.

Diameter was measured in cardiomyocytes with visible nuclei and intact cellular membranes. The width of individually isolated cardiomyocytes was manually traced across the middle of the nucleus, displayed on a viewing screen with a digitizing pad and determined by a computer-assisted image analysis system. Diameter was quantified in approximately 80 myocytes/rat, 3–4 rats/subgroup). Myocardial interstitial relative fibrosis area was also determined in 20 visual fields/rat. Collagen fibers were identified by the red-stained area and the collagen content was obtained by the ratio between the collagen fibers’ area and the total surface area. These analyses were carried out by ImageJ software (NIH, USA). The observer was blinded to the experimental groups.

### Electrophoretic gel mobility assay

LV nuclear extracts were prepared as previously described with minor modifications [27]. Double-stranded oligonucleotide containing the nuclear factor-kappaB (NF-κB, consensus sequence from Promega 5′AGTTGAGGGGACTTTCCCAGGC-3′), was end-labeled with γ-32P-ATP using T4 polynucleotide kinase. Unincorporated nucleotides were removed by passing the reaction mixture through a Sephadex G-25 spin column (Amersham-Pharmacia, Sweden). Purified 32P-labeled probe (30,000 cpm) was incubated in 20 μl with 10 μg of LV nuclear extracts in a binding reaction mixture containing 50 mM NaCl, 0.5 mM ethylenediaminetetraacetic acid (EDTA), 0.5 mM dithiothreitol (DTT), 4% glycerol, 10 mM Tris–HCl (pH 7.5) and 0.05 μg poly (dI-dC) for 30 min at room temperature. DNA–protein complexes were separated by electrophoresis through a 6% non-denaturing acrylamide:bis-acrylamide (37.5:1) gel in 0.5 × Tris-borate/EDTA for 2 h at 150 V. Gels were vacuum dried for 1 h at 80°C and exposed to X-ray film at −80°C. The bands were quantified by ImageJ software (NIH of Health, USA).

### Statistical analysis

Results are expressed as mean±SEM. Differences in treadmill performance between groups (SHR and WKY) and conditions were analyzed by 2-way ANOVA for repeated measurements (time). Differences in functional measurements, angiotensin content, oxidative stress, inflammatory profile, antioxidant defense and heart remodeling were analyzed by factorial ANOVA. Fisher was used as the post-hoc test. Differences were considered significant at P<0.05.

## Results

### Effects of aerobic training on treadmill performance and resting arterial pressure and heart rate in SHR and WKY

In spite of a better aerobic performance exhibited by the SHR (*vs*. age-matched controls) from the beginning of protocols, exercise training similarly increased the treadmill performance in both groups, with significant changes being observed after 4 and 8 weeks of training (Table 2). Notice that the performance gain between weeks 8 and 0 was similar in both strains. In contrast, SHR and WKY kept sedentary did not exhibit significant changes on attained treadmill speed after the 8 experimental weeks.

**Table 2:** Sequential changes on treadmill performance in normotensive (WKY) and spontaneously hypertensive rats (SHR) submitted to sedentary (S) or training (T) protocols.

	WKY-S	WKY-T	SHR-S	SHR-T
**Treadmill speed (km/h)**				
week 0	1.20±0.08	1.20±0.06	1.78±0.09*	1.79±0.06*
week 4	1.30±0.14	1.80±0.07†#	1.80±0.08*	2.30±0.06*†#
week 8	1.35±0.07	2.01±0.11†#	1.60±0.10*	2.70±0.13*†#
Performance gain (km/h)	0.07±0.04	+0.73±0.22†#	-0.20±0.17	0.85±0.18†#

Values are means ± SEM. Performance gain was calculated by the difference in attained velocity between weeks 8 and 0. For each strain (S+T) *n* corresponds to ~70–76 rats at week 0, ~36–40 rats at week 4, ~24–28 rats at week 8.

Significances (p < 0.05) are

* vs WKY.

† vs week 0.

# vs sedentary.

SHR were already hypertensive and exhibited increased HR at the beginning of protocols, showing an additional mean AP (MAP) increase during the 8-weeks sedentary protocol (Table 3). In the SHR, ageing-induced MAP increase was completely blocked by exercise training, which was accompanied by resting bradycardia, significant since the 4^th^ week (T_4_). Trained WKY only showed resting bradycardia from T_4_ up to T_8,_ without any change in MAP levels. (Table 3).

**Table 3:** Temporal changes on resting mean arterial pressure (MAP) and heart rate (HR) in normotensive (WKY) and spontaneously hypertensive rats (SHR) submitted to sedentary (S) or training (T) protocols.

Groups	MAP (mmHg)		HR (b.min^-1^)	
	WKY	SHR	WKY	SHR
T_0_ = S_0_	115 ± 1	162 ± 1*	330 ± 8	358 ± 7*
T_1_	112 ± 2	165 ± 3*	318 ± 4	347 ± 5*
T_2_	112 ± 2	165 ± 4*	317 ± 11	355 ± 4*
T_4_	114 ± 2	161 ± 4*	304 ± 8†	340 ± 7*†
T_8_	119 ± 1	161 ± 3*#	308 ± 7#†	326 ± 7*#†
S_8_	117 ± 2	187 ± 3*†	335 ± 11	402 ± 9*†

Values are means ± SEM. MAP and HR were measured in 8 to 10 animals/groups. Significances (p < 0.05) are

* vs WKY.

† vs week 0.

# vs S_8_.

### Temporal effect of aerobic training on plasma and heart angiotensin content in SHR and WKY

**Fig 1:**
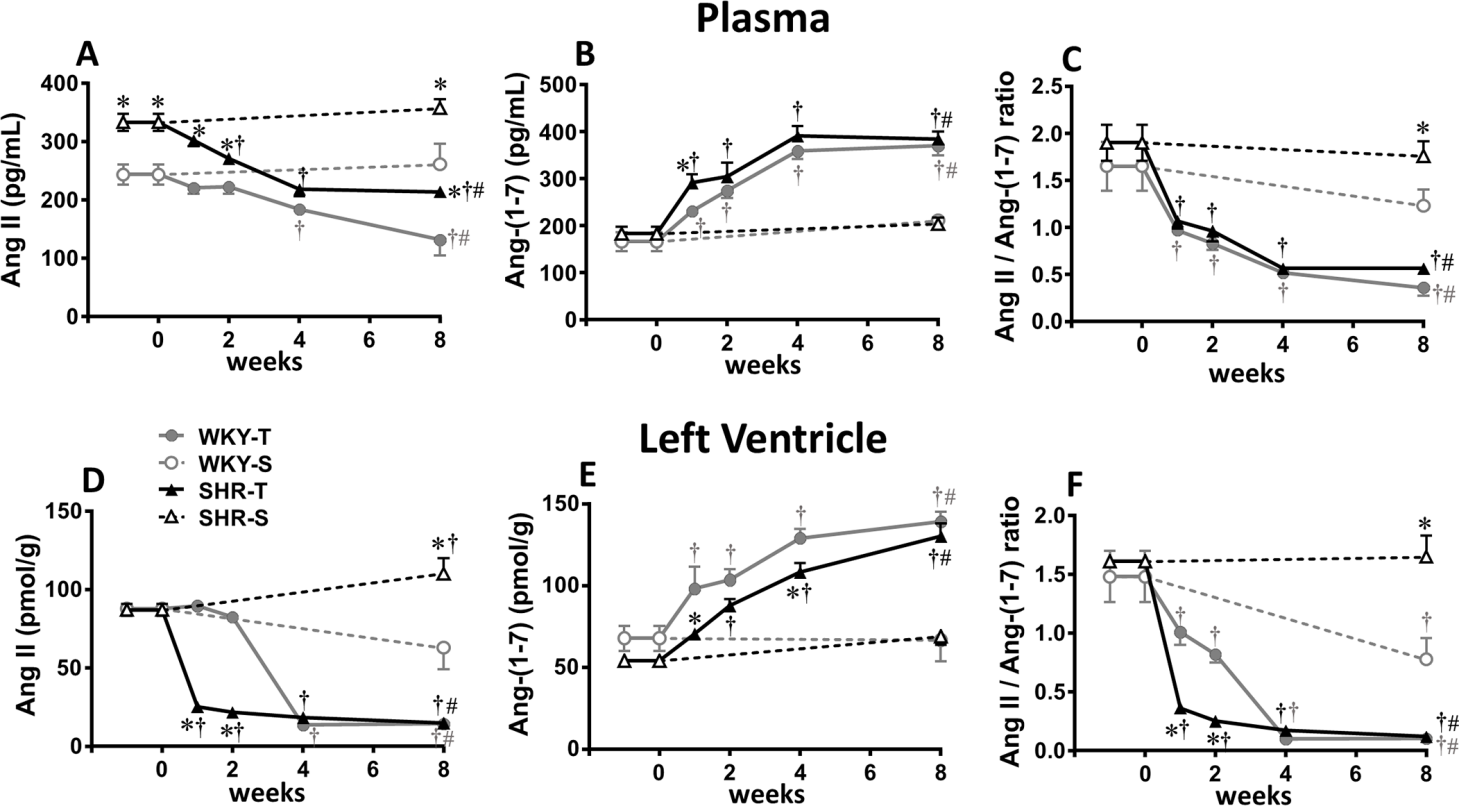
Training-induced changes in plasma and tissue angiotensin’s content. Time course changes of angiotensin II (Ang II, A and D) and angiotensin-(1–7) [(Ang-(1–7), B and E] content and their ratio (C and F) in the plasma (A, B, C) and left ventricle (LV, D, E, F) of sedentary (S) and trained (T) SHR and WKY during the 8 weeks’ protocols. Angiotensin peptides were measured in 6 to 8 animals/group. Significance is P<0.05 * *vs* WKY; † *vs* week 0; # T_8_ *vs* S_8_.

At the beginning of experiments, SHR-S when compared to age-matched controls exhibited high plasma Ang II content, but similar plasma Ang (1–7) levels (Fig 1A and 1B). At that time LV Ang II as well as Ang (1–7) content were similar between strains (Fig 1D and 1E). Except for the increased LV Ang II in the SHR-S, plasma and tissue content of angiotensin peptides was unchanged in both strains during the 8-weeks sedentary protocol. Training promptly reduced and nearly normalized LV (T_1_) and plasma Ang II (T_2_) of hypertensive rats, values that were maintained up to the end of the training protocol (Fig 1A and 1D). Training also reduced plasma and LV Ang II content of normotensive rats, but only after 4 weeks of exercise training. On the other hand, both SHR-T and WKY-T showed significant and similar increases of plasma and LV Ang (1–7) content (from the 1^st^-2^nd^ week on, Fig 1B and 1E). At the beginning of protocols, Ang II / Ang (1–7) ratio was similar for plasma and LV in both strains. However, it was higher in SHR *vs* WKY in sedentary groups at the 8^th^ experimental week (Fig 1C and 1F). Interestingly, while plasma Ang II / Ang (1–7) ratio was gradually and similarly reduced by aerobic training in both strains, LV Ang II / Ang (1–7) ratio was quickly reduced in the trained SHR (T_1_), a value attained by the WKY-T only after 4 weeks of training (Fig 1F). The prompt and specific reduction of Ang II content within the LV of the SHR suggests its possible causal effect in driving several beneficial adaptations induced by exercise training in the hypertensive heart [28–30].

### Temporal effects of aerobic training on heart oxidative stress in SHR and WKY

**Fig 2:**
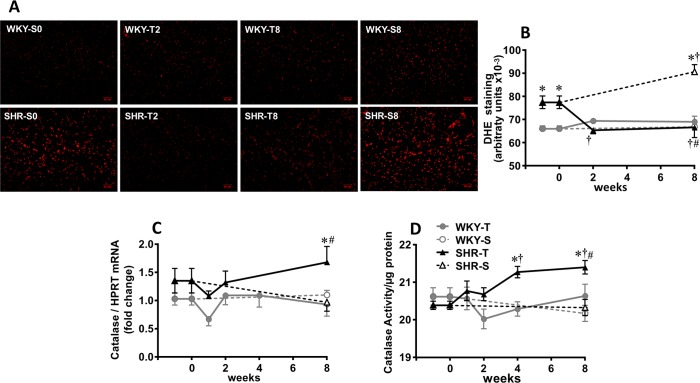
Sequential changes on heart oxidative stress and antioxidant defense. Temporal changes of reactive oxygen species (A, illustrative photomicrographs; scale bars = 50 μm; B, quantification of dihydroethidium staining, DHE) and catalase expression (C) and activity (D) in the left ventricle of sedentary (S) and trained (T) SHR and WKY during the 8 weeks’ protocols. DHE staining was measured in 4 rats/group (approximately 12–15 visual fields/rat); catalase expression and activity were evaluated in 4 to 6 rats/group. Significance is P<0.05 * *vs* WKY; † *vs* week 0; # T_8_ *vs* S_8_.

Although it is known that training effectively reduces both RAS and oxidative stress in several tissues [4,7,8,10,14], there is a paucity of information on the timing and/or volume of training for these effects to be established. Since heart Ang II hyperactivity was rapidly abrogated in the trained SHR, we next analyzed the time-course changes of reactive oxygen species (ROS, Fig 2A and 2B) within the LV. Dihydroethidium staining was significantly higher in the SHR-S_0_ when compared to WKY-S_0_ and continued to increase during the 8-weeks sedentary protocol. ROS bioavailability in the LV of trained SHR was markedly reduced and completely normalized at T_2_ (Fig 2B). No significant changes were observed in the LV dihydroethidium staining of the normotensive groups.

**Fig 3:**
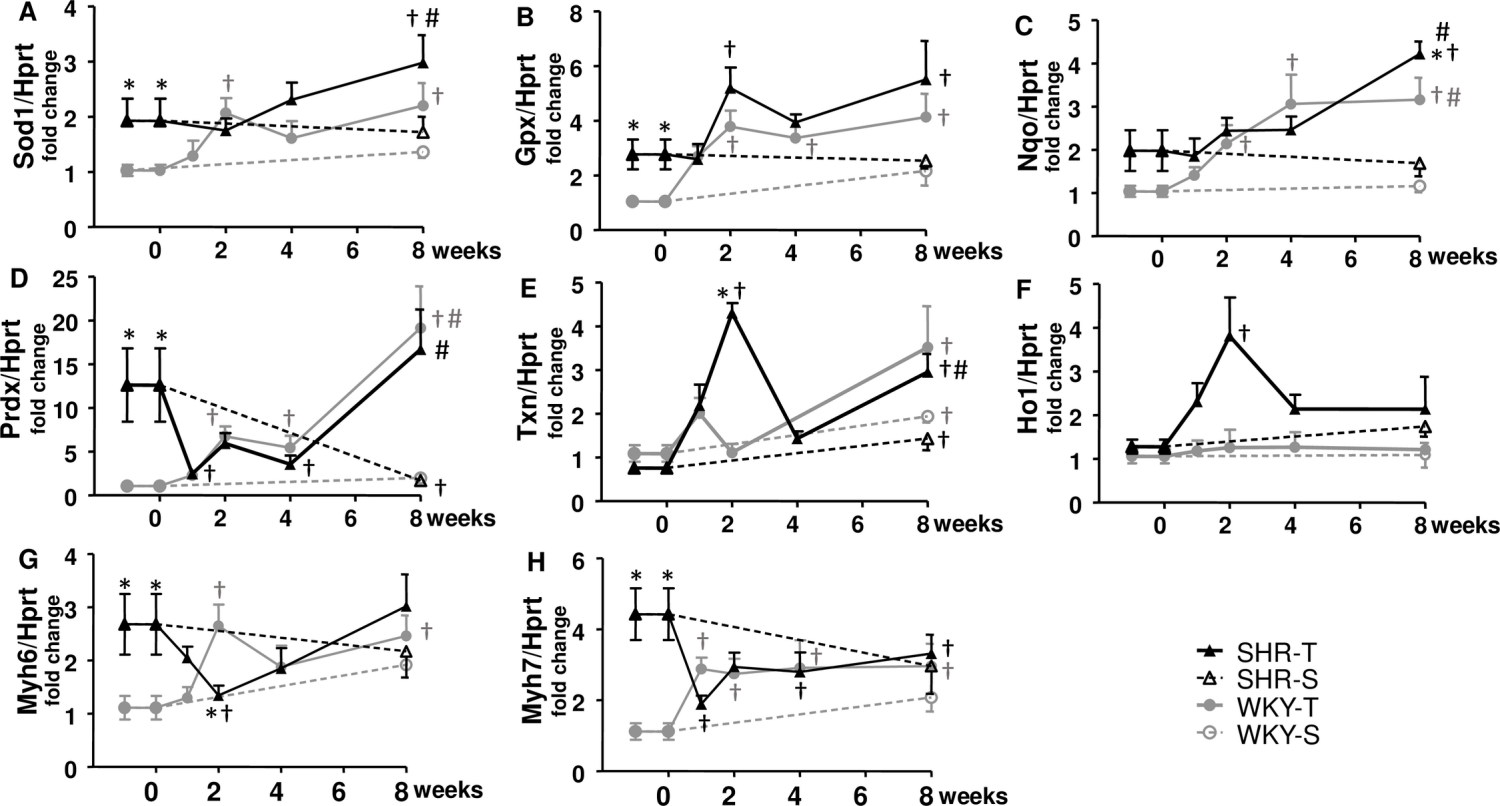
Temporal changes in left ventricle gene expression in normotensive (WKY) and spontaneously hypertensive rats (SHR) exposed to sedentary (S) or training (T) protocols. Superoxide dismutase 1 (Sod1, A), glutathione peroxidase (Gpx, B), NAD(P)H dehydrogenase quinone (Nqo, C), peroxiredoxin (Prdx, D), thioredoxin (Txn, E), hemioxigenase 1 (Ho-1, F), myosin heavy chain, α isoform (Myh6, G), myosin heavy chain, β isoform (Myh7, H) genes’ expression was measured in 4 to 6 rats/group. Significances (p < 0.05) is * vs WKY; † vs week 0; # vs S_8_.

Although recent studies have investigated antioxidant systems in hypertension and its role to oppose oxidative stress [31,32], very few is known about their response to exercise training in the heart of hypertensive individuals. Therefore, we also analyzed in the LV of both strains the effects of aerobic training on several antioxidant enzymes. At the beginning of protocols, SHR-S *vs*. WKY-S showed increased mRNA expression of superoxide dismutase, glutathione peroxidase and peroxiredoxin (Fig 3A, 3B and 3D) but similar expression of catalase, NAD(P)H dehydrogenase quinone, thioredoxin and hemioxigenase (Fig 2C and Fig 3C, 3E and 3F). Except for a significant reduction of peroxiredoxin mRNA in SHR-S_8_ and an increase in thioredoxin mRNA in both SHR-S_8_ and WKY-S_8_, no gene expression changes were observed in both sedentary groups. In contrast, training effectively augmented superoxide dismutase, glutathione peroxidase, NAD(P)H dehydrogenase quinone, thioredoxin and peroxiredoxin expression in both strains, but catalase mRNA expression only in the trained SHR (Fig 3A–3E and Fig 2C). The specific training-induced improvement of catalase expression in SHR was confirmed by the measurement of catalase activity in the LV: a significant increase was observed from T_4_ up to T_8_ (Fig 2D).

### Temporal effects of aerobic training on heart inflammatory profile in SHR and WKY

**Fig 4:**
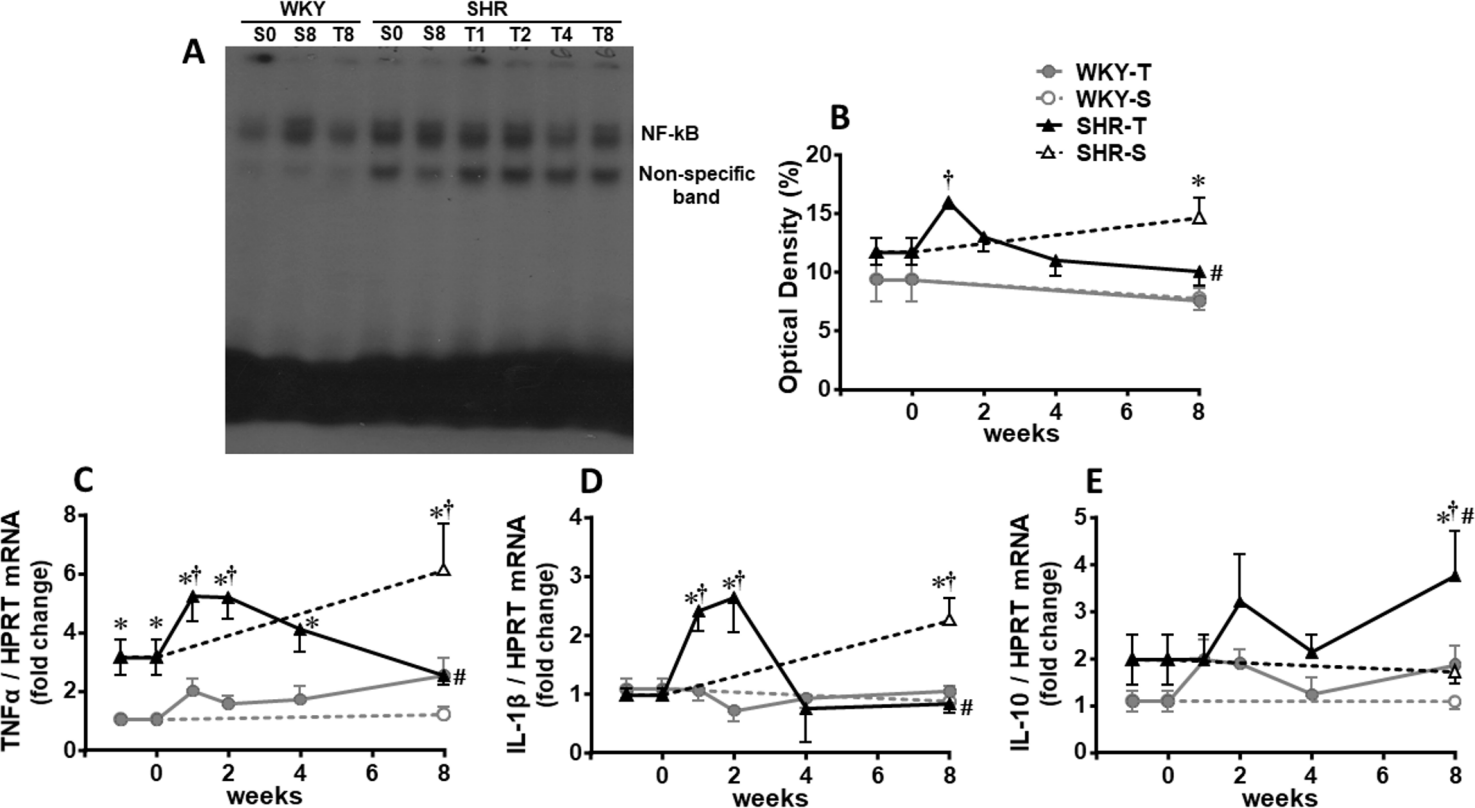
Training-induced changes on NF-kB, pro- and anti-inflammatory cytokines in the heart. Temporal changes of NF-kB translocation to the nucleus (A, gel shift assay for the p65 NF-kB monomer; B, measurements of optical density), TNF alpha (C), interleukin-1β (Il1b, D) and interleukin-10 (Il10, E) mRNA expression in the left ventricle of sedentary (S) and trained (T) SHR and WKY during the 8 weeks’ protocols. NF-kB translocation was measured in 5 rats/group and genes’ expression in 4 to 6 rats/group. Significance is P<0.05 * *vs* WKY; † *vs* week 0; # T_8_ *vs* S_8_.

Training reduces the low grade inflammation that characterizes hypertension [8,10,18]. Therefore, we sought to identify the temporal changes on pro-inflammatory profile of hypertensive rats (and their normotensive control) submitted to aerobic training. We analyzed the effects of training on both NF-kB translocation to the nucleus and the gene expression of pro-inflammatory and anti-inflammatory cytokines in the LV. At the beginning of the protocols, only TNF alpha was significantly elevated in SHR-S when compared to WKY-S (Fig 4C). In SHR kept sedentary, NF-kB showed a progressive increase during the 8 experimental weeks (Fig 4A and 4B), which was accompanied by a further increase in TNF alpha expression and a late but significant increase in interleukin-1β gene expression (Fig 4C and 4D). During the 1^st^ week of training, SHR showed a transient increase of NF-kB translocation to the nucleus simultaneously to the augmented gene expression of pro-inflammatory cytokines (T_1_-T_2_, Fig 4A–4D). From T_4_ up to T_8_, NF-kB activity was reduced and TNF alpha and interleukin-1β expression were back to control levels, attaining at the end of protocols values significantly lower than respective sedentary controls. Simultaneously with the reduced expression of pro-inflammatory cytokines, training induced a significant gene expression of interleukin-10 in the LV of trained SHR (T_8_, Fig 4E). Notice that no significant effects were observed in WKY rats submitted to the same training protocol (Fig 4A–4E).

### Temporal effects of aerobic training on cardiac remodeling in SHR and WKY

We also analyzed the time course changes induced by training on cardiac remodeling of hypertensive rats. Body weight and LV weight of the SHR and WKY during S and T protocols are presented in Table 4.

**Table 4:** Sequential changes on body weight (BW) and left ventricle (LV) weight in normotensive (WKY) and spontaneously hypertensive rats (SHR) submitted to sedentary (S) or training (T) protocols.

Groups	LV weight (mg)		BW (g)	
	WKY	SHR	WKY	SHR
T_0_ = S_0_	611 ± 30	768 ± 25*	255 ± 12	257 ± 7
T_1_	667 ± 21	776 ± 31*	270 ± 7	258 ± 7
T_2_	680 ± 26	813 ± 16*	283 ± 7	275 ± 5
T_4_	723 ± 18†	851 ± 15*†	297 ± 7†	290 ± 4†
T_8_	811 ± 13†	923 ± 34*†	333 ± 8†	303 ± 8*†
S_8_	792 ± 16†	919 ± 15*†	342 ± 7†	321 ± 4*†

Values are means ± SEM. LV weight and BW were measured in 8 to 10 rats/groups. Significances (p < 0.05) are

* vs WKY.

† vs week 0.

**Fig 5:**
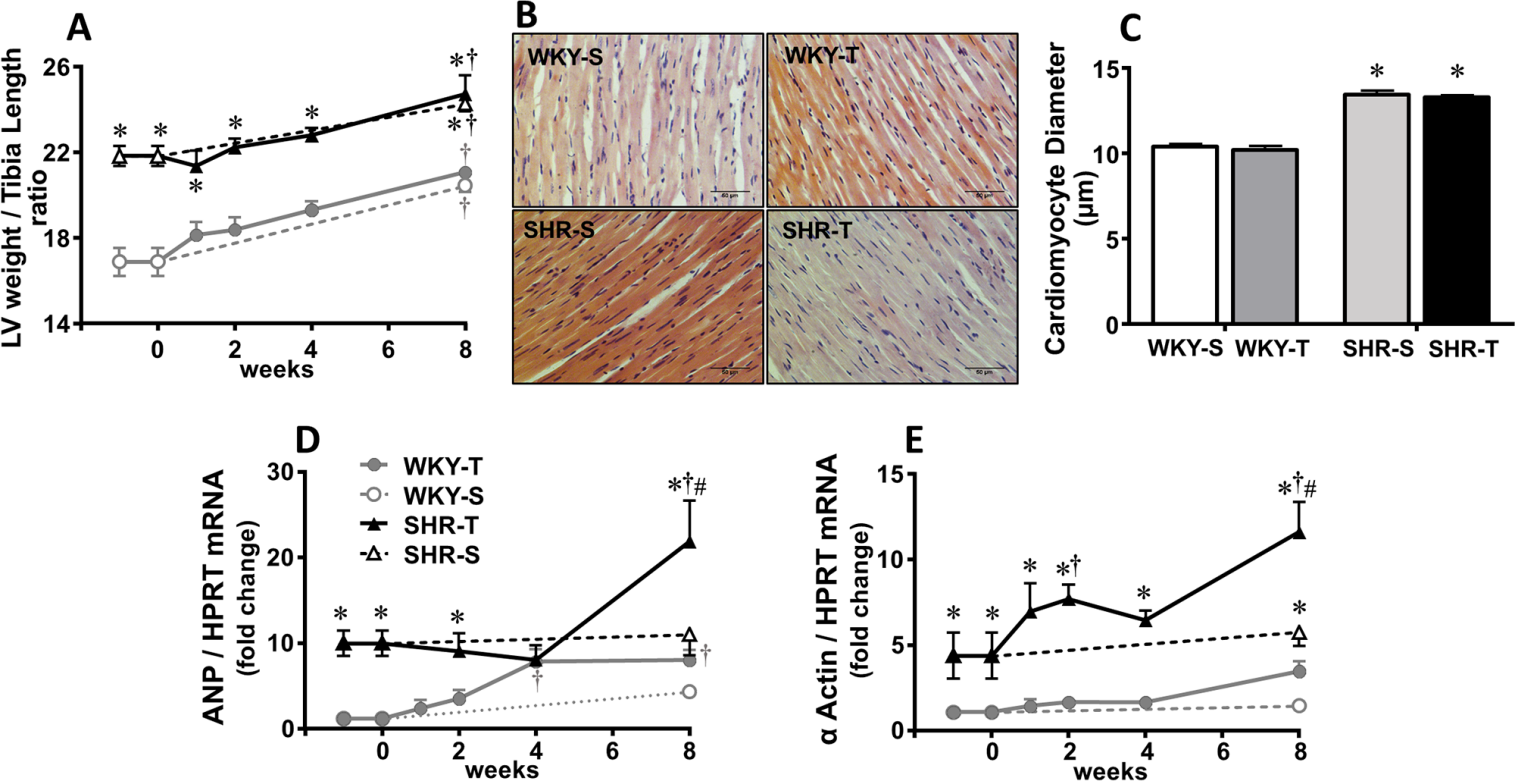
Sequential training-induced effects on cardiac remodeling. Time-related **c**hanges of left ventricle hypertrophy, as measured by LV/tibia length ratio (A), cardiomyocytes’ diameter at the end of protocols (B, illustrative photomicrographs, scale bars = 50μm; C, quantitative data), atrial natriuretic peptide (Nppa, D) and α-actin (Acta1, E) mRNA tissue expression in sedentary (S) and trained (T) SHR and WKY during the 8 weeks’ protocols. Cardiomyocytes’ diameter was measured in 3 to 4 rats/group (approximately 80 myocytes/rat) and gene expression in 4 to 6 rats/group. Significance is P<0.05 * *vs* WKY; † *vs* week 0; # T_8_ *vs* S_8_.

SHR-S when compared to WKY-S exhibited significant LV hypertrophy during the 8 experimental weeks, as indicated by LV weight/tibia length ratio (Fig 5A). In both strains, LV weight/tibia length ratio was even higher at the end of the 8 experimental weeks. These effects were confirmed by cardiomyocytes’ diameter measurement at the end of protocols (Fig 5B and 5C). Exercise training did not change these parameters.

**Fig 6:**
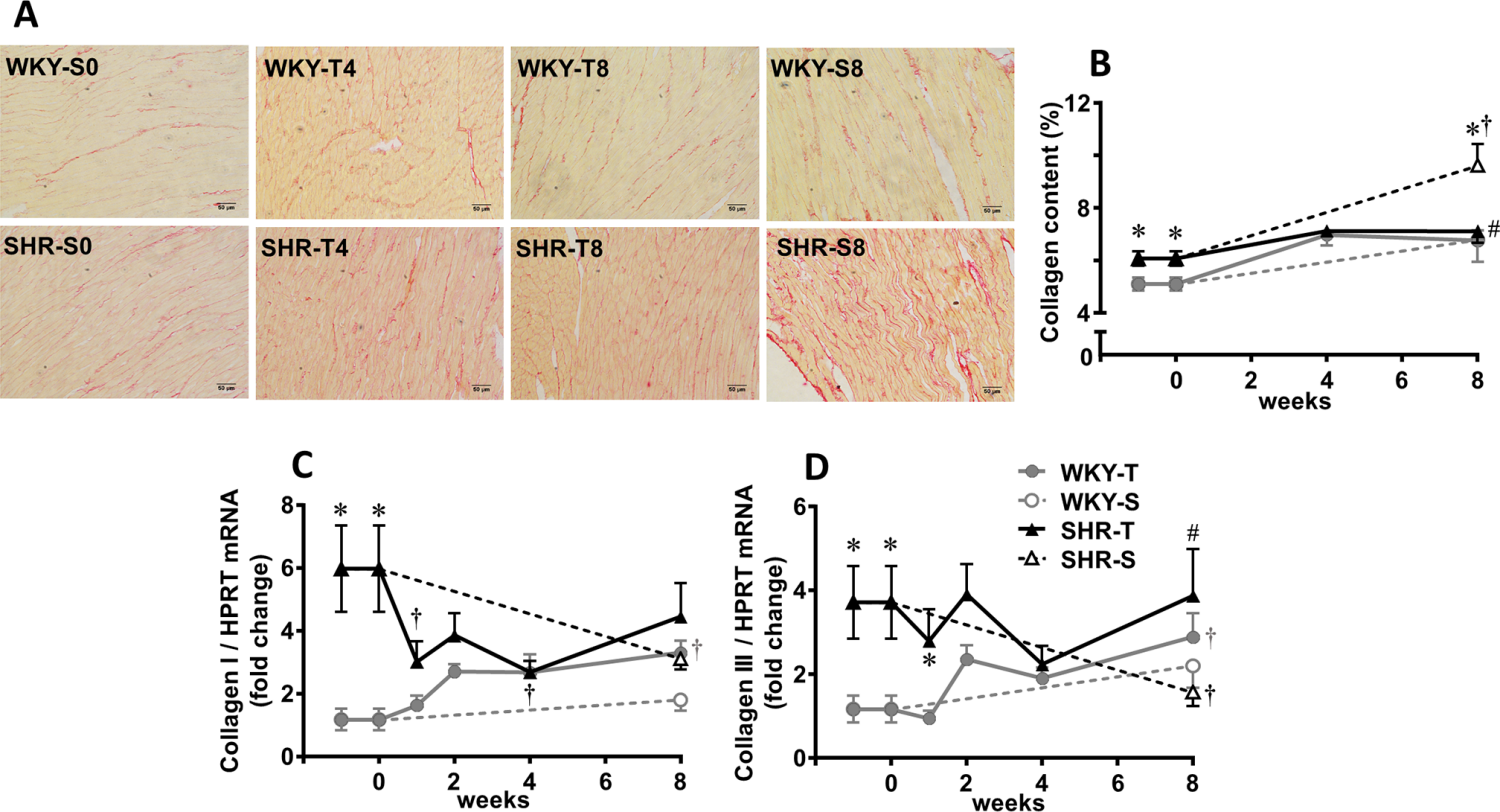
Training-induced changes on cardiac collagen expression. Changes of collagen content over time (A, illustrative photomicrographs, scale bars = 50μm; B quantitative data) and collagen I (C) and collagen III (D) mRNA expression in the left ventricle of sedentary (S) and trained (T) SHR and WKY during the 8 weeks’ protocols. Collagen content (picrossirius red) was measured in 3 to 4 rats/group (~20 visual fields/rat) and gene expression in 4 to 6 rats/group Significance is P<0.05 * *vs* WKY; † *vs* week 0; # T_8_ *vs* S_8_.

Gene expression of α and β isoforms of myosin heavy chain (Fig 3G and 3H), collagen I and collagen III (Fig 6C and 6D), atrial natriuretic peptide and α-actin (Fig 5D and 5E) were higher in SHR-S_0_ *vs* WKY-S_0_ and not significantly changed during the sedentary protocol, except by a small reduction in collagen I in SHR-S_8_. Exercise training caused a slight transient changes in mRNA expression of myosin heavy chain (both isoforms), but significant increases on atrial natriuretic peptide and α-actin expression in the LV of the trained SHR when compared to respective sedentary controls (Fig 5D and 5E). Transient changes with a tendency for collagen I and collagen III mRNA expression to decrease were observed in the trained SHR (Fig 6C and 6D). Measurement of collagen content within the LV confirmed these observations showing the ability of training to significantly reduce the collagen content in trained SHR (T_8_, Fig 6A and 6B). WKY-T also exhibited slight increases in gene expression of myosin heavy chain, atrial natriuretic peptide and collagen I and III during training, but no significant changes when compared to respective sedentary controls (Fig 3G and 3H, Fig 5D, Fig 6C and 6D). Coherently, no significant change on collagen protein expression was observed in the trained WKY.

## Discussion

Low-to-moderate exercise training is cardiovascular-protective and associated with reduced blood pressure in hypertension. Here we elucidate some mechanisms underlying these phenomena with focus on temporal relationships between the effects of exercise training on RAS activation, redox status and inflammatory responses in a rat model of spontaneous hypertension. Major findings from our study demonstrate that, i) reduced Ang II–ACE–AT_1_ receptor axis is the main effect to rapidly attenuate RAS hyperactivity in the heart; ii) this early withdrawal of the vasoconstrictor, pro-oxidative, pro-inflammatory and proliferative RAS axis is essential to counterbalance the initial transient exercise-induced increase of pro-inflammatory cytokines in the left ventricle of the trained SHR; iii) exercise training abrogates age-related translocation of NF-kB to the nucleus and the augmented expression of TNF alpha and interleukin-1β in the LV of sedentary SHR; iv) training augments cardiac antioxidant capacity, in part through increased catalase expression; and v) anti-oxidative and anti-inflammatory effects driven by aerobic training are accompanied by a significant reduction in cardiac collagen content without changing hypertension-induced left ventricle hypertrophy. In addition, our data show a different timing for plasma and heart hypertension-induced activation (increased circulating Ang II content precedes that of LV content) and training-induced deactivation (LV downregulation precedes plasma Ang II reduction) of the RAS axis.

The association between hypertension, RAS hyperactivity, oxidative stress and inflammation has been confirmed in experimental and clinical conditions [3,33–35]. Chronic hypertension is typically associated with cardiac hypertrophy and deleterious remodeling of the LV [8,13,21], processes that are Ang II-dependent. Most of these earlier studies focused on 1-time point to evaluate the cardiac status. Here we extend those studies, examining the processes involved in the development of hypertension and cardiac hypertrophy in response to exercise in a time- and age-dependent manner. Our data showed in sedentary SHR aged 3 months that hyperactivity of the vasoconstrictor axis of the circulating RAS (with a near normal vasodilator axis) preceded RAS activation in the LV, which only exhibited increased Ang II content (again with a normal Ang-(1–7) levels) in SHR aged 5 months. Precocious activation of the RAS has already been suggested by our previous findings showing significant increases of Ang II content in renal, carotid, femoral and thoracic aorta of the sedentary SHR aged 3 months [7]. Indeed, all RAS components have been shown to be independently expressed in different tissues as well as in the heart [3,12]. Here we revealed that high plasma Ang II and normal plasma and heart Ang (1–7) levels were kept constant from 3 up to 5 months of age, while LV Ang II augmented markedly in the sedentary SHR. This effect is accompanied by further increases in the oxidative stress, pro-inflammatory cytokines expression and collagen content, all them contributing to further LV hypertrophy observed in SHR from 3 up to 5 months. Our data also showed that these responses occurred simultaneously and should be related to Ang II changes since both plasma and LV levels of Ang-(1–7) were kept constant during the 8-weeks sedentary protocol.

Experimental and clinical studies suggest a relevant role for Ang II hyperactivity in hypertension-induced organ damage, since it is a potent vasoconstrictor, pro-oxidative, pro-inflammatory and proliferative stimulus [33,34]. Interestingly, besides the higher circulating Ang II content in the sedentary SHR at the beginning of protocols, tissue Ang II was not elevated in the hypertrophied LV, which already exhibited increased dihydroethidium staining, elevated TNF alpha expression and augmented collagen synthesis. Indeed, LV ROS production by Ang II-activated NOX complexes in cell membranes is not the only source for oxidative stress generation, since mitochondria, peroxisomes and endoplasmic reticulum are active sources as well [36]. On the other hand, activation of NAD(P)H oxidase by local Ang II seems to be responsible for the further increase in ROS bioavailability observed in the heart from 3 to 5 months, which was accompanied by additional increase in pro-inflammatory cytokines expression, collagen deposition and LV hypertrophy. As showed before, both Ang II and oxidative stress are potent stimuli for the low grade inflammation as well as collagen deposition [21,37], other important hallmark of hypertension.

Cardiac hypertrophy in the sedentary SHR was characterized by increased expression of fetal genes, increased expression of cardiac contractile proteins (with a larger increment in β than in α isoform of myosin heavy chain), augmented cardiomyocytes diameter and elevated collagen deposition, effects that occur early during cardiac remodeling. These hypertension-induced changes observed in SHR during development are similar to those previously described in the heart of ageing Sprague-Dawley rats and Long-Evans rats submitted to renovascular hypertension [38,39] and show comparable changes in cardiac contractile proteins and cellular responses [13,38]. We also observed that LV deleterious remodeling in the sedentary SHR was associated with increased antioxidant defenses (augmented superoxide dismutase 1, glutathione peroxidase and peroxiredoxin *vs*. WKY-S), which may reflect a compensatory response to pathological stress.

It is important to note that RAS, oxidative stress and pro-inflammatory cytokines act as a deleterious positive feedback cycle that would accelerate organ damage if therapeutic interventions are not provided [40]. In fact, several pharmacological therapies and life style changes have been employed to treat hypertension and ameliorate its adverse effects [17,35,41]. Exercise training, a non-pharmacological tool that partially reduce pressure levels has been shown to change the balance between vasoconstrictor and vasodilator RAS axes, to decrease both oxidative stress and pro-inflammatory profile and to minimize organ damage in hypertensive conditions [14,16,17]. However, observations made only at the end of experiments as well as the different training protocols used (type of exercise, duration, volume and/or training intensity) have generate some controversial results in the literature, most of them related to exercise-induced RAS responses [6,42,43]. This study comparing SHR and age-matched normotensive controls reported original data on time-course changes induced by low to moderate aerobic training on RAS peptides’ content in plasma and heart, as well as on sequential changes of LV oxidative stress, inflammation, remodeling and antioxidant defense. Our data confirmed previous observations that both deactivation of vasoconstrictor and facilitation of vasodilator axis contribute to training-induced Ang II/Ang-(1–7) ratio fall [6,42], revealing in addition that the downregulation of the ACE-Ang II-AT1 receptor axis in the trained SHR is the main effect to promptly knock down RAS hyperactivity in the heart (1^st^ week) and plasma (2^nd^ week of training). Similar, but late Ang II content reductions were also observed in the trained WKY (after the 4^th^ week of training for both plasma and LV content). On the other hand, significant and progressive augmentation of Ang-(1–7) since the 1^st^ week of training in plasma and LV occurred simultaneously in both groups, with a slightly lower LV response in the trained SHR. A recent review investigating the relation of acute and chronic exercise with blood pressure and RAS components confirmed our observation that training can stimulate ACE2-Ang-(1–7)-Mas receptor axis in parallel with the inhibition of ACE-Ang II-AT1 receptor pathway [44]. Notice that although Ang II/Ang-(1–7) ratio decrease was caused by changes in both vasoconstrictor and vasodilator axes, the marked and quick reduction of the vasoconstrictor axis was the main determinant of the prompt Ang II/Ang (1–7) ratio reduction, highlighting the important role of low-to-moderate exercise training in blocking the expression/activity of the ACE-Ang II-AT1 receptor RAS axis in the LV of the SHR. In addition, a recent study comparing low versus high volume of chronic aerobic exercise on RAS axes in a diet-induced obesity rat model showed that only the high, not the low volume exercise, was able to shift RAS balance towards the vasodilator axis [45].

The rapid training-induced reduction of Ang II/Ang (1–7) ratio was accompanied by a similar temporal decrease in ROS bioavailability in the LV. Our findings confirmed previous observations on the attenuation of oxidative stress by AT1 receptors blockade and Mas receptor activation [20] and on the decrease in the expression of gp91^phox^ and p47^phox^ NADPH-oxidase subunits by aerobic training [8,10]. They showed in addition that normalization of LV oxidative stress occurred in parallel with that of RAS changes, being complete in 2 weeks of training. Our data also confirmed previous observations that exercise training increases antioxidant enzymes’ expression [46–48], showing in addition that increased expression of antioxidant defense is a late effect only observed after 4–8 weeks of training. Interestingly, training augments gene expression of several antioxidant enzymes in both strains, but increased catalase expression (gene and protein) is a specific training-induced antioxidant defense observed only in the LV of the SHR. Notice that with exception of the catalase response to exercise, our results on antioxidant defense were based on gene expression data. Further studies are necessary to confirm the effects of training on protein expression and/or antioxidant enzymes activity.

Single bouts of exercise increase redox signaling and inflammation in skeletal muscle and heart while regular exercise decreases both [49–51]. However, the transition from the acute response to chronic adaptations has not been investigated. Our temporal data in trained and sedentary SHR revealed that the acute inflammatory effect of exercise (transient increases of TNF alpha and interleukin-1β expression in the LV mediated by NF-kB translocation to the nucleus) persisted during the first 2 weeks of training, coming back to control levels from weeks 4 to 8. In contrast, sedentary SHR showed a progressive age-dependent increase of the LV inflammatory profile. Therefore, at the end of the 8 experimental weeks, trained SHR, when compared to sedentary age-matched controls, exhibited lower levels of pro-inflammatory cytokines, which were associated with a significant training-induced increase in interlrukin-10, an important anti-inflammatory cytokine. Another original observation arising from this temporal set of data is that the transient exercise-induced inflammatory surge is counterbalanced by the robust withdrawal of the pro-oxidative and pro-inflammatory RAS axis observed since the 1^st^ week of training.

The present results indicated that the low-to-moderate exercise training protocol is cardio-protective by downregulating the RAS and reducing oxidative injury, but did not reverse the cardiac hypertrophy exhibited by SHR rats. Indeed, a meta-analysis on the effects of exercise on cardiac hypertrophy confirmed the absence of training effects in SHR hearts when the exercise protocol started in adult rats aged 3-months or older [52]. Previous studies also demonstrated different remodeling responses to treadmill exercise, wheel running and swimming training [13,19,42,52,53]. In our hands, treadmill training stimulated the expression of fetal genes and reduced the collagen content within the LV of the SHR, without changing gene expression of myosin heavy chain, cardiomyocytes’ diameter and the age-induced increase in LV/tibia length ratio. It should be noted that although higher expression of atrial natriuretic peptide is an index of pathological hypertrophy [13], lower levels (as those stimulated by physiological stimulus like the low-intensity exercise training) may act as a protective factor. Supporting this idea, it was already demonstrated that when binding to its receptor (NPR-A), atrial natriuretic peptide antagonized the deleterious effects induced by both cardiac overload and Ang II [54].

Taken together our data show that exercise training does not reverse hypertension-induced cardiac hypertrophy, but rapidly decreases local synthesis of Ang II (T_1_), normalizes the oxidative stress (T_2_), augments antioxidant defense (T_4_-T_8_) and reduces both inflammatory profile (T_8_) and collagen deposition (T_8_) in the LV, partially reversing the deleterious cardiac remodeling exhibited by the SHR. Cardiac Ang II changes precede training-induced reduction in plasma Ang II content (T_2_-T_4_) and pressure fall (T_8_). These findings indicate that early downregulation of ACE-Ang II-AT1 receptor RAS axis is a crucial mechanism to improve cardiac function in the trained SHR, showing in addition this effect occurs before training-induced plasma RAS deactivation.
